# Generation of TIL-based Cellular Products for Cancer Immunotherapy: Current Insights and the Challenges

**DOI:** 10.32607/actanaturae.27559

**Published:** 2025

**Authors:** D. V. Kuznetsova, T. V. Petrova

**Affiliations:** Lopukhin Federal Research and Clinical Center of Physical–Chemical Medicine, Moscow, 119435 Russia

**Keywords:** tumor-infiltrating T lymphocytes, immunotherapy, T-cell therapy, TIL

## Abstract

Tumor-infiltrating T lymphocytes (TILs) are a population of T cells present in tumor tissue and enriched in tumor antigen-specific clones. TILs participate in the adaptive antitumor immune response, which makes them a promising candidate for cancer immunotherapy. The concept framing this type of therapy involves the extraction of T cells from a patient’s tumor, followed by their in vitro expansion and reinfusion into the same patient in large quantities. This approach enhances the antitumor immune response and allows one to affect cancer cells resistant to other types of treatment. In 2024, the first TIL-based drug was approved for melanoma treatment. The possibility of using TILs for treating other solid tumors is currently being considered, and novel methods aiming to increase the efficiency of generating TIL cultures from tumor tissues in vitro are being developed. However, despite the significant progress achieved in this area, there remain unresolved issues and problems, including the lack of standardized protocols for obtaining, expanding, and cryopreserving TILs, the complexity related to their isolation and the duration of that, as well as insufficient efficiency. Our review focuses on the concept of immunotherapy using TILs, the main stages involved in generating a TIL-based cellular product, associated problems, and further steps in the production of TIL cultures that aim to improve efficiency as relates to production and ensure a wider application of the therapy.

## INTRODUCTION

Cancer immunotherapy is among the most innovation-prolific and promising areas of modern oncology. As new data on the interplay between the immune system and tumors become available, various forms of immunotherapy (therapy using immune checkpoint inhibitors, including antibodies specific to molecules such as CTLA-4, PD-1, PD-L1, etc.; CAR T cell therapy, CAR NK cell therapy; dendritic cell therapy; in vitro generation followed by reinfusion of autologous tumor-infiltrating T lymphocytes (TILs) back into the patient’s body; and vaccination with chemically synthesized neoantigen peptides) has started to be viewed as a promising novel approach to the treatment of different types of malignant tumors, since it allows practicians to personalize treatment and improve its efficacy even in patients with uncontrolled and metastatic cancer.

Currently, cancer immunotherapy is fertile ground for the research and development of novel drugs.

Tumor-infiltrating T lymphocytes are a population of T cells within tumor tissue that are enriched in tumor-specific clones. However, the immunosuppressive factors inherent in the tumor microenvironment actively suppress the antitumor immune response and weaken the ability of TILs to destroy tumor cells. The concept of TIL therapy is based on the idea that the antitumor immune response can be restored by isolating TILs from a tumor fragment, culturing them ex vivo to increase their quantity (to at least 109 cells), and finally reinfusing them back into the patient. Unlike other cell-based immunotherapy methods, TILs are obtained directly from the patient, without any genetic modification [1].

The research in the 1950s that aimed to explore how possible it was to employ T cells to suppress tumor cell growth was inspired by studies that had demonstrated that rejection of solid organ transplants was mediated by cellular immunity [2]. Animal experiments showed that when transferred to syngeneic recipients, T cells from immunized donors could mediate tumor regression, and that IL-2 could be used to increase their number [3]. Later, it was revealed using a mouse model that simultaneous administration of IL-2 and T cells in vivo enhances the antitumor efficacy of T cells. However, the requirement that an immunized syngeneic donor be the source of the tumor-specific T cells remained a hurdle in attempts to use this approach in humans lacking such a source of TILs.

This hurdle was overcome in 1986, when Rosenberg and colleagues from the Surgery Branch of the National Cancer Institute (USA) became the first to demonstrate, in a mouse model, that a combination of autologous TILs and cyclophosphamide could induce a regression of metastases [4]. Next came a landmark publication in 1988 that became the first study to show that infusion of TILs into patients with metastatic melanoma could lead to tumor regression [5]. As of October 2024, a total of 266 clinical trials related to TIL therapy had been registered on ClinicalTrials.gov. Of those, 26 trials have the "active" status; 103 trials are "recruiting participants," and 82 trials have been completed [6]. Over the past five years, 15–30 new clinical trials to assess TIL therapy against various solid tumors have been registered annually, melanoma being predominant (40% of all clinical trials) [7].

**Table 1:** Selected clinical trials of TIL therapy registered on Clinicaltrials.gov as of October 2024

Nosological entity	NCT identifier number	Phase of clinical trial	Number of patients	Administered dose
Stage IIIb, IIIc or IV melanoma	NCT03374839	I/II	11	Cohort 1: 5 × 10^8^ TILs (three patients)/ Cohort 2: 1–20 × 10^9^ TILs on weeks 14 and 18
Stage IV melanoma	NCT03475134	I	10	N/A
Measurable metastatic melanoma	NCT03166397	II	30	N/A
Unresectable stage III/IV melanoma or platinum-resistant ovarian cancer	NCT03158935	Ib	24	1 × 10^10^–1.6 × 10^11^ TILs
Unresectable stage III/IV cutaneous or mucosal melanoma	NCT02652455	Pilot	12	N/A, cell growth after 4–8 weeks when using
CD137-activating antibody Measurable metastatic melanoma	NCT02621021	II	170	N/A, young TILs
Unresectable metastatic melanoma	NCT02360579	II	60	N/A
Metastatic melanoma or stage III in-transit, subcutaneous, or regional nodal disease	NCT01740557	Pilot	15	Up to 1.5 × 10^11^ TILs
Unresectable stage III/IV melanoma	NCT02354690	I/II	12	1 × 10^9^–2 × 10^11^ TILs
Unresectable stage III/IV melanoma	NCT02278887	III	168	N/A
Metastatic melanoma or stage III in-transit, subcutaneous, or regional nodal disease	NCT01955460	Pilot	15	Up to 1.5 × 10^11^ TILs
Metastatic melanoma	NCT01993719	II	64	N/A
Unresectable stage III or IV melanoma	NCT01946373	I	10	Up to 5 × 10^10^ TILs
Unresectable stage III/IV melanoma	NCT01883323	II	12	1 × 10^10^ –1.6 × 10^11^ TILs
Metastatic melanoma, uveal melanoma or stage III in-transit or regional nodal disease	NCT00338377	II	189	Cohort 1–3: up to 1.5 × 10^11^ TILs. Cohort 4: 5.0 × 10^9^ TILs on day 1, 10 × 10^10^ TILs on day 15
Metastatic uveal melanoma	NCT03467516	II	59	1 × 10^9^–2 × 10^11^ TILs
Metastatic melanoma NCT01995344 II 90 N/A Unresectable stage III/IV melanoma	NCT02379195	I/II	12	N/A
Stage III/IV melanoma	NCT01807182	II	13	N/A
Unresectable melanoma, stage III/IV	NCT01701674	Pilot	13	N/A
Unresectable stage IV metastatic melanoma or stage III in-transit or regional nodal disease	NCT01659151	II	17	N/A
Metastatic melanoma	NCT01319565	II	102	Cohort 1 + 2: 1 × 10^0^ –2 × 10^11^ young TILs
Unresectable stage III/IV melanoma	NCT01005745	I/II	19	N/A
Locally advanced, recurrent, or metastatic biliary tract cancer	NCT03801083	II	59	2 × 10^11^ TILs (at least 1 × 10^9^ cells)
Metastatic uveal melanoma	NCT03467516	II	47	2 × 10^11^ TILs (at least 1 × 10^9^ cells)
Breast cancer	NCT05142475	I	50	1 × 10^9^–5 × 10^10^ TILs
Malignant solid tumors	NCT05649618	I	42	2.5 × 10^9^–5 × 10^10^ TILs
Advanced solid cancers	NCT03935893	II	240	2 × 10^11^ TILs (at least 1 × 10^9^ cells)
Malignant solid tumors	NCT05902520	I	18	N/A
Urothelial cell carcinoma (UCC) and non-muscle invasive bladder urothelial carcinoma (NMIBC)	NCT05768347	I	12	N/A
Advanced melanoma	NCT05098184	I	50	1 × 10^9^–5 × 10^10^ TILs
Metastatic III and IV stage melanoma	NCT01883323	II	12	1.0 × 10^6^ cells/mL and expanded for no longer than 28 days prior to cryopreservation
Melanoma	NCT02360579	II	66	26.1 × 10^9^ (range, 3.3–72) TILs
Non-small cell lung cancer	NCT04614103	II	170	1 × 10^9^–150 × 10^9^ TILs
Cervical cancer	NCT03108495	II	27	28 × 10^9^ TILs

Table 1 presents a selective list of phases I and II clinical trials that embrace nearly all solid tumor types.

On February 16, 2024, the FDA approved lifileucel (Amtagvi), the result of 30 years of research, as the first TIL-based therapeutic. The drug was approved for adult patients with unresectable or metastatic melanoma who had previously received standard treatment. Lifileucel is produced through ex vivo cultivation of tumor-infiltrating T lymphocytes derived from surgically resected autologous tumor fragments [8].

Metastatic melanoma is considered a highly immunogenic malignant tumor. The objective response rate to TIL therapy ranges from 36 to 56%; progression-free survival is 3.7–7.5 months; the overall survival time ranges from 15.9 to 21.8 months [9]. Less immunogenic (also known as "cold") tumors respond worse to TIL therapy, which poses a problem on one hand, while, on the other hand, it opens up new avenues towards developing new strategies to optimize TIL-based treatments.

## EX VIVO PRODUCTION AND EXPANSION OF TUMOR-INFILTRATING T LYMPHOCYTES

**Fig. 1:**
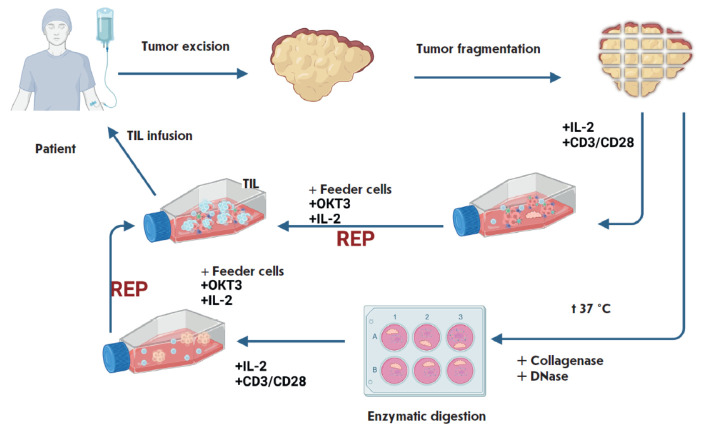
Preparation and infusion of TILs obtained from freshly resected tumor tissue. The two most common options are shown: obtaining TILs from tumor fragments and by enzymatic digestion. Regardless of the type of pre-REP stage of TIL production, at the second stage (REP), feeder cells need to be added to ensure large-scale expansion before infusing the cellular product into the patient

Ex vivo expansion of tumor-infiltrating T lymphocytes can be divided into two stages: the production of TIL cultures from tumor tissue (the pre-REP stage) and large-scale expansion of T cells (the REP stage) (Fig. 1).

Freshly resected tumor tissue obtained during a surgical resection is promptly transported to the laboratory within several hours after surgery in a sterile container with the transport medium (a growth medium supplemented with an antibiotic). The biological material is immediately cut into small fragments sized approximately 1.5 × 1.5 mm² and placed into a growth medium supplemented with interleukin-2 (IL-2) at concentrations ranging from 500 to 6,000 IU/mL. An alternative method for TIL culture generation involves enzymatic digestion of tumor fragments in an enzyme cocktail containing collagenase and DNase at 37°C for 30–60 min. The resulting cell suspension is subsequently transferred to a growth medium supplemented with IL-2 (500–6,000 IU/mL) [10, 11, 12, 13]. To further activate TIL cultures, IL-2 is used in combination with anti-CD3/CD28 antibodies in some protocols [14, 15, 16, 17, 18]. Since clinical research includes studying the feasibility of producing TILs from tumors of different localizations, including skin and gastrointestinal tumors, one should bear in mind that bacterial contamination of tumor fragments is possible. Therefore, additional washing steps and/or ex vivo cultivation in the presence of antibiotics and antifungals are recommended to mitigate this risk. Some protocols also involve pre-incubation of tumor fragments in a medium containing 10% antibiotics at room temperature for 30 min prior to further manipulations, in particular when working with colorectal cancer or melanoma specimens [19]. The initial stage is considered completed once the cell count in the primary TIL culture reaches ~ 106 cells per mL of suspension. Next, TILs may either undergo cryopreservation or one can proceed to the second stage: large-scale expansion (REP) aiming to generate a clinically significant number of cells. According to clinical trials and the instruction for use of the approved medicinal product lifileucel, the number of TILs required for infusion ranges from 1 × 10^9^ to 2 × 10^11^ cells; the total infusion volume being 100–400 mL [12, 20]. Feeder cells (either peripheral blood mononuclear cells from healthy donors (allogeneic) or from the patient (syngeneic)) pre-irradiated with 40 Gy are utilized for large-scale expansion during the second phase. The feeder cells are cocultured with TILs in a growth medium containing IL-2 (500–6,000 IU/mL) until the clinically significant number of TILs is reached [19]. Since effective ex vivo TIL expansion largely depends on the number of feeder cells, standard protocols recommend using the 100 : 1 or even 200 : 1 ratio of feeder cells to TILs [21]. It is commonly believed that fewer feeder cells can significantly reduce the yield of TILs, thus underscoring their importance for successful expansion [15]. Because large quantities of feeder cells need to be utilized, clinicians often use donor-derived feeder cells and pool material from multiple donors [12]. This approach is unique to TIL therapy compared to the more common method used for producing CAR T cells, where feeder cells are not employed. Instead, high doses of IL-2 and anti-CD3/CD28 activating antibodies are simultaneously added directly to the growth medium to stimulate T-cell proliferation [22, 23].

## EFFECTIVENESS IN THE GENERATION OF TUMORINFILTRATING T LYMPHOCYTES

As mentioned previously, reliable generation of TIL cultures from tumor tissue fragments is the cornerstone of successful TIL therapy. An analysis of the studies conducted by various researchers (Table 2) revealed that the likelihood of obtaining viable TIL cultures from patients’ tumor fragments is weakly dependent on the type of solid tumor and varies across study sites. TIL cultures were successfully generated in 18–100% of patients across the studies. The findings summarized in Table 2 infer that this variability is partly attributable to the lack of standardization for the TIL culture generation procedures, as well as to the fact that certain tumor types (e.g., colorectal cancer and melanoma) carry a higher risk of microbiological contamination. Furthermore, factors such as the quantity of initial tumor biomaterial and the degree of immune cell infiltration into it (as observed in uveal melanoma and glioblastoma) play a rather significant role. Unfortunately, the small sample size in most studies weighs negatively on the integrity and validity of the reported data and may lead to both over- and underestimation of the effectiveness of TIL culture generation. We have found just one study that focused on effectiveness in TIL culture generation in a large patient cohort (over 1,000 subjects). It could be inferred from the results of the study that the effectiveness varied by year; over an 11-year period, TIL cultures were produced in an average of < 70% of patients [33].

**Table 2:** The features and effectiveness of generating TIL cultures from solid tumors

Nosological entity	State of the initial tumor tissue sample	Number of tumor tissue samples in the study	Features of TIL culture generation	Effectiveness of TIL culture generation, %	Percentage of CD4^+^, CD8^+^ T- cell populations of the total cell count in the TIL culture, %	Percentage of contaminated TIL cultures	Reference
Melanoma	Freshly resected material	90 tumors, 710 individual cultures	1) TILs cultured from tumor fragments. Growth medium: RPMI 1640, 100 U/mL penicillin, 100 μg/mL streptomycin, 2 mmol/L L-glutamine, 10% human serum, IL-2 (6,000 IU/mL). 2) TILs from enzymatically digested tumor fragments. Solution for enzymatic digestion: collagenase, hyaluronidase, and DNase in RPMI 1640. 18-hr incubation of the fragments on an orbital shaker. Culturing the resulting cell suspension in the TIL growth medium.	1) 69.9 2) 94.1	CD4^+^ 31.4 (0.3–70), CD8^+^ 62.4 (37.2–97.6)	N/A	[12]
Breast cancer	Freshly resected material	42 tumors	N/A	100	CD4^+^ 55.6, (9.1–94.0)	N/A	[24]
Colorectal cancer, stomach cancer	Freshly resected material	33 colorectal tumors, 8 stomach tumors	TILs from enzymatically digested tumor fragments. Four-week culturing of tumor fragments.	Colorectal cancer, 64 Stomach cancer, 43	N/A	N/A	[25]
Colorectal cancer	Fresh material	12 tumors	TILs cultured from tumor fragments. Growth medium: CellGenix GMP DC, 10% human serum, 1% solution of an antibiotic antifungal agent, IL-2 (1,000 IU/mL). 10 ng/mL IL-12 was added when transferring the TIL culture to a perfusion bioreactor for largescale expansion.	100	First phase of expansion: CD4^+^ 28.8 (0.6–55.3), CD8^+^ 64.6 (325–84.5). During the second phase of expansion: CD8^+^ 85, CD4^+^ 12.4 (1.7–40.5).	N/A	[26]
Uveal melanoma	Freshly resected material	30 tumors	1) TILs cultured from tumor fragments (22 tumors). Culturing 1–4 tumor fragments in 2 mL of the TIL growth medium containing IL-2. Haft of the growth medium was replaced with fresh one every 2–3 days. 2) TILs from enzymatically digested tumor fragments (12 tumors). Solution for enzymatic digestion: collagenase D 10 mg/mL, DNase I 3 mg/mL. 30-min incubation on a GentleMACS dissociator. 3) TILs from enzymatically digested tumor fragments with additional positive CD3 selection using Dynabeads magnetic beads (25 tumors).	1) 18 2) 42 3) 68	CD4^+^ 25 (0–91), CD8^+^ 39 (6–84)	N/A	[27]
Soft tissue sarcoma	Freshly resected material	64 tumors	1) TILs cultured from tumor fragments. Culturing one tumor fragment per well of a 24-well plate in the growth medium containing IL-2 (6,000 IU/mL). 2) TILs from enzymatically digested tumor fragments. Solution for enzymatic digestion: DNase IV (30,000 U/L), hyaluronidase V (100 mg/L), collagenase IV (1,000 mg/L), gentamicin (500 mg/L), penicillin-streptomycin (5,000 U/mL), L-glutamine (292 mg/L), amphotericin B (62.5 μg/L). Culturing cell suspension in the growth medium containing IL-2 (6,000 IU/mL).	91	CD8^+^ 54.2 (3–95.4), CD4^+^ 2.5 (0.03–44.73)	1.60	[28]

## NOVEL APPROACHES TO THE GENERATION OF TUMOR-INFILTRATING T LYMPHOCYTES

Despite significant progress, effectiveness in TIL culture generation remains well below 100%. Moreover, TIL cultures must be enriched with cytotoxic CD8^+^ T cells to ensure an optimal antitumor response in vivo. Meanwhile, Table 2 suggests that the proportion of CD8^+^ T cells greatly varies and is potentially affected by both the initial ratio of T cells within the tumor tissue and the specific culture conditions.

Current research focuses on optimizing protocols for TIL culture generation by supplementing the growth medium with various interleukin cocktails, utilizing genetic engineering at different T-cell production stages, and working with the immunosuppressive tumor microenvironment, which can ruin the full potential of antitumor cellular therapy.

One of the approaches to enhancing effectiveness in ex vivo generation of TIL cultures from tumor tissue involves adding immune checkpoint modulators into the growth medium. Several research groups have demonstrated that adding an agonistic anti-4-1BB antibody to melanoma tissue fragments reduces the expansion duration and increases the proportion of CD8^+^ T cells within the TIL culture compared to a conventional growth medium containing IL-2 only [35, 36]. Similar effects by this antibody have been observed in 16 samples of non-small cell lung cancer. A combination of IL-2 and agonistic anti-CD3 and anti- 4-1BB antibodies (urelumab) added to the TIL culture medium reduced the time required to generate TIL cultures and increased the proportion of CD8^+^ T cells during both the pre-REP and REP stages of TIL culturing in [37]. This approach ensured 100% effectiveness during TIL culture generation for 12 uveal melanoma samples [33]. Since uveal melanoma is characterized by a low immune cell infiltration, TIL culture generation from this tumor type poses a significant challenge. The number of TILs obtained in this study from five fragments less than 3 mm³ in size was comparable to, or exceeded, that produced from 20 fragments using the conventional method (IL-2 only) [33].

Another approach to the interplay with the tumor microenvironment was proposed by a research team that had demonstrated the effectiveness of inhibiting the prostaglandin E2 (PGE2) signaling pathway to stimulate an antitumor response in vivo [38]. Relying on these findings, Morotti et al. discovered that effectiveness in TIL culture generation from melanoma samples (NCT03475134) could be improved by inhibiting the PGE2 signaling pathway. Inhibition of this signaling pathway increased the susceptibility of TILs to IL-2, thus reducing the impact of oxidative stress on T cells and their ferroptosis-mediated death [39].

Addition of various interleukin cocktails to the growth medium is another promising approach to enhancing effectiveness in TIL culture generation, including the production of cultures exhibiting tailored properties (e.g., TIL cultures with a predominant proportion of CD8^+^ T cells or cultures enriched in memory T cells rather than effector T cells). The application of interleukin cocktails involves a move away from the conventional use of IL-2 alone for T-cell activation and allows one to study how different cytokines (IL-4, IL-7, IL-15, and IL-21) and their combinations affect the end cellular product. Cytokine cocktails had originally been widely used to culture another cellular product: CAR T cells [40, 41, 42]. In further studies, CAR T cells cultured in media supplemented with IL-7 and IL-15 exhibited higher proliferation rates and enhanced antitumor activity compared to cells cultured just in the presence of IL-2 [43]. Furthermore, it has been established that adding a combination of IL-2, IL-15, and IL-21 increases the CD8^+^/CD4^+^ T-cell ratio [44], which is especially important in CAR T therapy.

Because of the successful application of various interleukin combinations in CAR T therapy, similar approaches are now being adopted for the generation of TIL-based products. Studies involving PD-1+CD8^+^ T cells isolated from the blood of healthy donors and patients with a confirmed diagnosis of cancer demonstrated that a cytokine cocktail containing IL-7 and IL-15 added to the growth medium, along with anti- CD3/CD28 antibodies, significantly enhances T-cell proliferation in the suspension [45]. Treatment with a combination of anti-CD3 antibodies, panobinostat, IL-2, and IL-21 was shown to increase the proportion of CD62L+CD28+CD8^+^ T cells in TIL cultures compared to TILs cultured in the absence of this cytokine cocktail [46].

The research into the use of interleukins to enhance T-cell expansion continues to advance; modified forms of interleukin are being actively developed. For example, a genetically engineered IL-2 (STK-012) is currently under development; it is the first-in-class partial agonist of the IL-2 receptor alpha and beta chains (IL-2Ra/β) required to selectively activate CD25+ antigen-activated T cells without inducing the nonspecific activation of NK cells or naïve T cells. Preclinical in vivo studies in mice using the murine surrogate mSTK-012 revealed a significant reduction in the number of exhausted T cells and increased systemic and intratumoral expansion of the tumor antigen-specific CD25+PD-1+CD8^+^ T cell population. Additionally, the number of intratumoral regulatory T cells (Tregs) was decreased, indicating that mSTK-012 exhibits better antitumor properties compared to those of IL-2 [47, 48].

Orthogonal cytokine–receptor pairs for human IL-2 that interact exclusively with each other have been developed when studying interleukin modifications. Notably, these pairs do not interact with their native counterparts: cytokine IL-2 and its receptor IL-2. Introduction of orthoIL-2Rβ into the T-cell suspension has enabled selective targeting of orthoIL-2 to genetically modified CD4^+^ and CD8^+^ T cells, both in vitro and in vivo. This approach can reduce adverse events and minimize toxicity compared to that of the canonical form of IL-2 [49].

The next, potential candidate modifier of the antitumor activity of T cells is interleukin-12 (IL-12), a pro-inflammatory cytokine that plays a crucial role in the activation of CD4^+^ and CD8^+^ T cells, as well as NK cells. The high toxicity of IL-12 has been limiting its clinical application. Preclinical studies suggest that the toxicity of IL-12 is primarily associated with the activation of NK cells. An attempt was made to address this problem using an IL-12 partial agonist (STK-026), which has reduced affinity for binding to the IL-12 receptor β1 subunit (IL-12Rb1). STK-026 selectively affected activated T cells characterized by upregulated IL-12Rb1 expression, whereas NK cells or resting T cells with moderate IL-12Rb1 expression levels were not significantly affected by STK-026 [50]. The Synthekine company is currently conducting preclinical trials for STK-026, which are expected to demonstrate its capacity to activate tumor-infiltrating CD8^+^ T cells and myeloid cells, as well as its antitumor efficacy and pharmacodynamic profile.

As mentioned previously, genetic modification of T cells is a possible path in addressing the problem of efficient TIL culture generation and enhancement of their functionality.

Recent studies have shown the great potential that lies in engineering T cells carrying an inducible membrane-bound IL-12. These modified T cells exhibited superior cytotoxic activity in vitro and were characterized by a significant level of IFN-γ production [51].

Obsidian Therapeutics, a pharmaceutical company, is currently involved in a multicenter clinical trial to evaluate potential uses for genetically modified TILs OBX-115 expressing membrane-bound IL-15 (mbIL15). This approach allows one to avoid in vivo administration of high-dose IL-2, thereby reducing the toxicity and expanding the applicability of TIL therapy to larger patient cohorts [52].

Rejuvenation of tumor-infiltrating T lymphocytes is another interesting strategy for augmenting their antitumor activity using genetic engineering means. This approach allows for the rejuvenation of TILs by restoring their original functionality and potential via partial reprogramming using transient expression of a set of transcription factors. The rejuvenated TILs retain a diverse repertoire of their T-cell receptors (TCRs), thus ensuring broad antigenic specificity. The key positives of TIL rejuvenation consist in a reduction of the epigenetic age of T cells, higher expansion rates, acquisition of a stem cell phenotype, and increased cytokine secretion upon activation by target antigens. Importantly, positive results have been achieved not only for rejuvenated TILs but also for rejuvenated peripheral blood mononuclear cells (PBMCs), TCR and CAR T cells, which indicates that the rejuvenation technology can be widely applied in cancer immunotherapy [51].

Developing vectors for the in vivo delivery of genes to modifying tumor-specific T cells is the last aspect of gene engineering discussed in this review. The technique aims to optimize TIL therapy. Current research in designing viral vectors for in vivo gene delivery focuses on restricting viral tropism to specific T-cell markers such as CD3, CD8, CD4, CD62L, and CD5 [53, 54, 55]. Thus, the efficacy of retroviruses targeting the peptide–MHC complex (pMHC) for delivering genes, including interleukin-12, to antigen-specific T cells and promoting their in vivo expansion, was evaluated in a recently published preprint. Preliminary results of mouse experiments demonstrate that pMHC-targeted viruses are effective vectors for the reprogramming and expansion of tumor-infiltrating T lymphocyte populations in vivo.

The reviewed studies demonstrate that diverse approaches are being pursued to optimize the production of T cell-based products, which will broaden the range of their clinical applications [56].

## CRYOPRESERVATION IN THE GENERATION OF TUMOR-INFILTRATING T LYMPHOCYTES

The previously mentioned cryopreservation of TILs is highly desirable; in certain cases, it is essential both for manufacturing and in cases when TILs need to be reinfused back into the patient after some time. Cryopreservation implies the slow freezing of cellular products at a rate of ~ 1°C per min in a growth medium containing cryoprotectants, dimethyl sulfoxide (DMSO) being the most commonly used, followed by storage in liquid nitrogen until the product is needed. However, cryopreservation adversely affects all cellular products, including TILs, altering the cytokine production, cytotoxic activity, proliferation, and cell viability [17].

Meanwhile, the therapeutic efficacy of cellular products is directly dependent on the ability of the cells to restore their viability and functionality following thawing.

Although current FDA protocols for both TIL therapy [57] and CAR T therapy [58] permit the use of both fresh cellular products and cryopreserved ones, research into the activity of T cell-based products is ongoing, since the post-thaw viability and functionality of T cells is far from ideal. Importantly, unlike for CAR T cells, the proportion of antigen-specific T cells within the T cell-based product is relatively low, ranging from 0.1 to 9% [59]. Therefore, any reduction in the number of viable cells following the freeze–thaw cycle can critically affect the quality of the T cell-based product. Because the TIL therapy is such a novel technique, very little data on the effects of cryopreservation on TIL quality is available. Three patents have been approved so far. They focus on the optimization of TIL cryopreservation [60 ,61, 62]. TILs cryopreserved after the pre-REP stage have also been used to produce cellular products in the phase I clinical trial NCT03215810 to assess the TIL therapy in patients with lung cancer [63]. Additionally, as mentioned previously, the approved drug lifileucel is supplied in cryopreserved form, in accordance with the manufacturer’s recommendations.

Data on the impact of cryopreservation on CAR T-cell therapy, which has been in clinical use for an appreciably long time, appear somewhat scattered. Based on the information of some CAR T-cell manufacturers, the post-thaw viability ranges from 47.2 to 68.9% [64]. Conversely, another research group has reported an average viability of 97 ± 17.4% in previously cryopreserved CAR T-cell fractions. A total of 79 ready-to-use CAR T infusion products where CAR T cells were expanded to a median value of ~ 1 × 10^6^ cells per kg of body weight (range, 1 × 10^5^ to 1 × 10^7^ cells/kg) were analyzed. The median cryopreservation duration was nine days (range, 1–408 days). Despite the high survival rates in this case, the thawed CAR T cells exhibited increased expression of early apoptotic markers [65]. Another study demonstrated that cryopreservation during the expansion phase does not hinder cell proliferation post-thaw; CAR T cells continued to divide in 86% of cases [66]. Additionally, the study that examined the stability of cryopreserved CAR/TCR T-cell controls showed that these cells remained stable for at least one year after thawing. After 12 months, the viability of thawed cells stood at approximately 80%, remaining stable for at least six hours post-thaw [67].

In an assessment of the tolerance of peripheral blood lymphocytes to cryopreservation following large-scale expansion in the presence of high-dose IL-2, the T cells immediately lost their ability to respond to nonspecific stimulation with phytohemagglutinin after thawing. However, their reactivity was restored within 48 h. Cell viability remained high (> 80%) throughout this process, although each subsequent cryopreservation cycle resulted in a loss of approximately 10–15% of the cells [68].

Comparative analysis with other types of immune cells indicates that regulatory T cells (Tregs) and NK cells also exhibit poor cryopreservation tolerance. One day post-thaw, the proportion of viable NK cells decreased from 64–91% to ~ 34% [69]. A similar trend was observed for Tregs: the percentage of live cells immediately after thawing ranged from 58 to 75%, declining to 20–48% after 24 h [70].

A potential solution towards improving the viability of T cells after cryopreservation is to directly cryopreserve tumor fragments [71, 72, 73, 74, 75]. A recent study focusing on the isolation of tumor-infiltrating T lymphocytes from frozen colorectal cancer tissue fragments demonstrated that the efficiencies of TIL culture generation from individual aliquots of cryopreserved fragments of the same tumor were similar after thawing and analyses at different time points, thus indicating data reliability. Furthermore, similar CD4^+^/CD8^+^ T-cell ratios were observed in TIL cultures derived from both frozen and fresh tumor fragments [76]. A comparative analysis of TIL generation from fresh vs. frozen tumor samples showed that, although initial expansion occurred at a faster pace in fresh tissue, the total number of viable cells equalized approximately after one week of culturing [77]. In an Australian study where fresh and cryopreserved melanoma fragments derived from the same patients had been transported to a laboratory for further TIL expansion for four days, only the cryopreserved fragments ensured a 100% rate of successful culture generation [78]. Furthermore, in one patent, no phenotypic differences between TILs derived from fresh vs. frozen tumor tissues were listed [59]. Hence, the use of cryopreserved tumor fragments is a viable strategy that allows one to preserve the source of TILs for subsequent expansion, thus addressing the logistical challenges related to the transportation of biological material from the hospital where the tumor had been excised to manufacturing sites, including remote ones. However, standardization is needed for cryopreservation of expanded TILs, as well as tumor fragments and possibly new cryopreservation media, which would improve TIL survival and efficiency in generating TIL-based cellular products.

## CONCLUSIONS

Immunotherapy that utilizes tumor-infiltrating T lymphocytes shows great potential as relates to the treatment of various types of cancer. Characterized by a unique specificity to tumor-associated antigens, TILs can effectively destroy malignant cells, especially in melanoma, where this therapy has already proven to be effective.

Despite the encouraging preliminary results, TIL-based therapy is still in its infancy. Some unresolved issues related to therapeutic effectiveness across different tumor types persist, and there exists no standardized protocol for the isolation, expansion, and cryopreservation of TILs. In order to improve therapeutic effectiveness, research aiming to develop unified protocols and optimize the processes related to current challenges is needed.

An important area of focus is exploring novel strategies to augment the antitumor immune response that would be specifically aimed at overcoming the immunosuppressive microenvironment within tumors. Achieving these goals will encourage broader application of TIL-based therapy and improve prognosis for patients with various cancers.

## Abbreviations

TIL: tumor-infiltrating T lymphocytes
CTLA-4: cytotoxic T lymphocyte-associated protein 4
PD-1: programmed cell death protein 1
PD-L1: programmed death-ligand 1
CAR: chimeric antigen receptor
CAR-T: chimeric antigen receptor to T cells
CAR-NK: chimeric antigen receptor-engineered natural killer
TCR: T-cell receptor
FDA: Food and Drug Administration
pre-REP: pre-rapid expansion protocol
REP: rapid expansion protocol
PGE2: prostaglandin E2
IL-2: interleukin-2
IL-2Ra/β: interleukin- 2 receptor alpha and beta chain
ortho-hIL-2: orthogonal human genetically engineered interleukin-2
ortho-hIL-2Rβ: orthogonal human genetically engineered interleukin-2 receptor
IL-7: interleukin-7
IL-12: interleukin-12
IL-12Rb1: interleukin-12 receptor beta 1 subunit
IL-15: interleukin-15
mbIL15: membrane-bound interleukin-15
IL-21: interleukin-21
4-1BB (CD137 or TNFRSF9): member of the tumor necrosis factor receptor family
IFN-γ: interferon gamma
PBMC: peripheral blood mononuclear cell
DMSO: dimethyl sulfoxide
Tregs: regulatory T cells
NK cells: natural killer cells
MHC: major histocompatibility complex
pMHC: major histocompatibility complex peptide
